# Protein Kinase G Is Involved in Acute but Not in Long-Term Regulation of Renin Secretion

**DOI:** 10.3389/fphar.2019.00800

**Published:** 2019-07-18

**Authors:** Andrea Schramm, Frank Schweda, Maria Luisa S. Sequeira-Lopez, Franz Hofmann, Peter Sandner, Jens Schlossmann

**Affiliations:** ^1^Institute of Pharmacy, Department of Pharmacology and Toxicology, University of Regensburg, Regensburg, Germany; ^2^Institute of Physiology, University of Regensburg, Regensburg, Germany; ^3^University of Virginia School of Medicine, Charlottesville, VA, United States; ^4^Institute of Pharmacology and Toxicology, Technical University of Munich, Munich, Germany; ^5^Bayer AG, Drug Discovery—Cardiology, Wuppertal, Germany

**Keywords:** PKG, cGK, cGMP, sGC stimulation, renin, RAAS, juxtaglomerular apparatus

## Abstract

Pharmacological inhibition of the renin–angiotensin–aldosterone system (RAAS) is, in combination with diuretics, the first-choice treatment for hypertension, although 10–20% of patients do not respond adequately. Next to the RAAS, the nitric oxide/cGMP/protein kinase G (PKG) system is the second fundamental blood pressure regulator. Whether both systems influence each other is not well-studied. It has been shown that nitric oxide (NO) supports renin recruitment *via* activation of soluble guanylate cyclase (sGC) and subsequent generation of cGMP. Whether this leads to an ensuing activation of PKGs in this context is not known. PKGIα, as well as PKGII, is expressed in renin-producing cells. Hence, we analyzed whether these enzymes play a role regarding renin synthesis, secretion, or recruitment. We generated renin-cell-specific PKGI-knockout mice and either stimulated or inhibited the renin system in these mice by salt diets. To exclude the possibility that one kinase isoform can compensate the lack of the other, we also studied double-knockout animals with a conditional knockout of PKGI in juxtaglomerular cells (JG cells) and a ubiquitous knockout of PKGII. We analyzed blood pressure, renin mRNA and renal renin protein content as well as plasma renin concentration. Furthermore, we stimulated the cGMP system in these mice using BAY 41-8543, an sGC stimulator, and examined renin regulation either after acute administration or after 7 days (application once daily). We did not reveal any striking differences regarding long-term renin regulation in the studied mouse models. Yet, when we studied the acute effect of BAY 41-8543 on renin secretion in isolated perfused kidneys as well as in living animals, we found that the administration of the substance led to a significant increase in plasma renin concentration in control animals. This effect was completely abolished in double-knockout animals. However, after 7 days of once daily application, we did not detect a persistent increase in renin mRNA or protein in any studied genotype. Therefore, we conclude that in mice, cGMP and PKG are involved in the acute regulation of renin release but have no influence on long-term renin adjustment.

## Introduction

The NO/cGMP system and the renin–angiotensin–aldosterone system (RAAS) are both crucial players in blood pressure (BP) regulation and can be seen as two scale pans in BP balance. While NO/cGMP acts vasodilatory *via* different mechanisms (reviewed in Thoonen et al., 2013), the RAAS is essential for an upregulation of BP. The juxtaglomerular cells (JG cells) are the exclusive site for secretion of renin in the kidney and consists of myofibroblast-like cells that are located in the media layer of vasa afferentia at the entrance into the glomerulus. Because renin is the key enzyme in the RAAS, the regulation of renin secretion is of great importance in the regulation of this system and occurs, on the one hand, *via* on-/offset of renin synthesis and, on the other hand, *via* control of renin secretion. Moreover, during chronic challenges of the RAAS, the kidney reacts *via* hypertrophy of juxtaglomerular cells and *via* transformation of preglomerular vascular smooth muscle cells (VSMCs) into renin-producing cells, the so-called “renin recruitment” (Owen et al., 1995; Sequeira López, et al., 2004). Because upregulation of plasma renin concentration (PRC) leads to hypertension and organ damage in humans, it would be of great importance to understand the underlying signaling mechanisms that trigger renin synthesis, secretion, and recruitment. cAMP is one of the best investigated second messengers in this context. This molecule plays a fundamental role in control points of renin activity regarding regulation of renin gene transcription and renin secretion. Regarding modulation of renin synthesis, it is commonly accepted that transcriptional regulation *via* different regions within the *renin* gene is the most essential step (Pan and Gross, 2005; Martinez et al., 2018). Among others, the cAMP/CRE/CREB system is an important transcription factor system involved in renin gene regulation (Pan et al., 2001). Next to cAMP, the cGMP signaling pathway is also indispensable for gene regulations. Recently, it has been shown that cGMP-dependent protein kinase Iα (PKGIα) mediates GATA4 (a transcription factor which is important for cardiac development and diseases) transcriptional activity by phosphorylation and physical association of both interaction partners (Ma et al., 2016). Whether cGMP/PKG also affects renin gene transcription is only partially characterized so far (Wagner et al., 1998).

The next step in renin synthesis following transcription and translation is the maturation of preprorenin to prorenin, which is then sorted in the Golgi apparatus and either directly released into circulation or further processed to the enzymatically active renin. For that to happen, glycosylation steps are necessary, which in turn lead to a direction of glycosylated prorenin into the vesicular network in the JG cell. A final proteolytic cleavage gives rise to mature renin, a protein with a molecular mass of around 36 kDa, which is then stored in intracellular vesicles until the cell obtains a release signal (Yokosawa et al., 1980). The underlying mechanisms of these processes as well as the identity of the protease responsible for renin activation still remain unclear (Gross et al., 2010).

Renin secretion occurs in response to various signals, e.g., catecholamines or prostaglandins, and is basically mediated through an increase in cAMP (likely by subsequent activation of PKA) (Friis et al., 2002). However, contradictory data were published concerning nitric oxide (NO) and thus cGMP involvement in renin secretion. In the 1990s, NO was thought to inhibit renin release (Beierwaltes and Carretero, 1992; Salazar et al., 1992; Sigmon et al., 1992). Later, more and more convincing evidence was supplied, which supports the view that the NO/cGMP system is a positive regulator of renin secretion (Tharaux et al., 1997; Beierwaltes, 2006; Schnermann and Briggs, 2012). Moreover, NO, its intracellular receptor soluble guanylate cyclase (sGC), and cGMP have a prominent role in renin recruitment: in 2013, Neubauer et al. showed that endothelial NO synthase (eNOS)-produced NO supports renin cell recruitment and, more prominent, that this process is dependent on cGMP generated by sGC (Neubauer et al., 2013). To date, four different effectors for cGMP are known: cyclic nucleotide gated channels (CNG channels), phosphodiesterases (PDEs), the multidrug resistance protein 4 (MRP4), which transports cGMP/cAMP out of the cells (van Aubel et al., 2002), and cGMP-dependent protein kinases (PKGs). In JG cells, it is known that cGMP interferes with cAMP degradation by inhibition of phosphodiesterase 3 (PDE3), thereby enabling cGMP/cAMP crosstalk. The subsequent increase in cAMP concentration leads to exocytosis of renin storage granula so that NO/cGMP indirectly supports renin secretion *via* PDE (Kurtz et al., 1998; Beierwaltes, 2006; Sällström et al., 2010). Whether PKGs also take part in this regulation or if they can mediate renin cell recruitment is not known. These kinases exist in three isoforms: PKGIα, PKGIβ, and PKGII (Pfeifer et al., 1999). PKGIα and β are expressed from the same gene and differentiated through alternative splicing and are localized in the cellular cytoplasm (Wernet et al., 1989). Besides the investigation of expression patterns in other tissues (summarized in Geiselhöringer et al., 2004), we focused on renal distribution of PKGI in the past years and detected, next to other kidney parts, a prominent expression of PKGIα (but not PKGIβ) in the juxtaglomerular cell (Schinner et al., 2013). In the present study, we tested the hypothesis, whether this kinase is also involved in renin regulation. As experiments with global PKGI-knockout animals are rather difficult due to their reduced life span and severe phenotype including elevated blood pressure (Pfeifer et al., 1998), we generated renin-cell-specific conditional PKGI-knockout mice to address the function of this kinase regarding renin regulation. Next to PKGIα, PKGII is also expressed in JG cells; former reports suggested that renin secretion is inhibited by PKGII (Gambaryan et al., 1996; Wagner et al., 1998). To rule out the possibility that one kinase can compensate the lack of the other, we also conducted experiments with animals missing both kinases (global PKGII knockout, renin-specific PKGI knockout). By administration of different salt diets as well as ACE inhibition by enalapril, we challenged the RAAS in these animals and checked the capability of the system to react to these stimuli. Furthermore, we stimulated the cGMP system by Bay 41-8543, an sGC stimulator, to study the effect of cGMP kinase deletion on the renin system.

## Material and Methods

### Animals

All animal experiments were performed in accordance with the Guidelines for the Care and Use of Laboratory Animals published by the US National Institutes of Health and approved by the local authorities for animal research (Regierung von Unterfranken, D-97064 Würzburg, Germany; #RUF-55.2-2532-2-515-21). Renin cell-specific PKGI knockouts were derived from crossings of mice bearing a heterozygous insertion of Cre recombinase in the Ren1d gene locus (Ren1d^Cre/+^) on a 129SVxC57/Bl6 background [kindly provided by ML Sequeira López and R. Ariel Gomez (Sequeira López, et al., 2004)] and mice carrying a floxed exon 10 of the PKGI-gene (PKGI^fl/fl^) (Wegener et al., 2002). Crossing of these mice resulted in Ren1d^Cre/+^-PKGI^flfl−^ animals (considered as Ren-PKGI-KO). Ren1d^+/+^-PKGI^fl/fl^ animals were considered as controls (hereinafter referred to as Ctr). The Ren1d^+/+^-PKGI^fl/fl^ or Ren1d^Cre/+^-PKGI^fl/fl^ animals were crossed with PKGII-knockout mice (Pfeifer et al., 1996) to obtain finally either PKGI fl/fl/PKGII −/− (considered as PKGII-KO) or Ren-Cre/PKGI fl/fl/PKGII −/− (considered as Ren-PKGI-KO/PKGII-KO) animals. Genotyping was performed by PCR on DNA isolated from tail or ear biopsies (Renin-Cre: mRen-Ren: 5’-GAAGGAGAGCAAAAGGTAAGAG-3’, mRen Cre: 5’-TTGGTGTACGGTCAGTAAATTGGAC-3’; PKGI-flox: RF125: 5’-GTCAAGTGACCACTATG-3’, RF53: 5’-CCTGGCTGTGATTTCACTCCA-3’; PKGII-KO: AV3R: 5’-ATTAAGGGCCAGCTCATTCC-3’, AV9R: 5’-CTGCTTAATGACGTAGCTGCC-3’, E2FBAV: 5’-GGTGAAGTTTTAGGTGAAACCAAG-3’). Experimentation was conducted in male 8–40-week-old mice and litter-matched controls. Mice were housed in a 12:12-h light–dark cycle and kept on standard rodent chow with free access to tap water. In addition, for stimulation of renin recruitment, mice were either receiving low salt diet (LS, 0.03% NaCl; Ssniff, Germany) for 10 days or LS+enalapril (LS/Ena, 10 mg/kg/day in drinking water; Sigma-Aldrich, Munich, Germany) for 3 weeks. To inhibit RAAS, mice were kept on high salt chow (HS; 4% NaCl; Ssniff, Germany) for 3 weeks. For stimulation of cGMP signaling, BAY 41-8543 was administered i.p. for either 45 min or for 7 consecutive days once daily [1 mg/kg/day, dissolved in vehicle (0.6 g glycerol/1 g H_2_O/9.49 g PEG 400); Bayer Pharma AG, Wuppertal, Germany]. Mice receiving only vehicle served as controls.

### Blood Pressure Measurement

Systolic blood pressure of conscious mice was determined by tail cuff plethysmography (IITC Life Science, Woodland Hills, USA). Mice were trained for at least 3 consecutive days; after that, approximately 30 measurements were recorded, and values were averaged over 5–10 representative readings.

### Isolated-Perfused Kidney

Kidneys of mice of different genotypes were perfused *ex situ* at a constant perfusion pressure (100 mmHg) as described previously (Schweda and Kurtz, 2004). The basic perfusion medium contained a modified Krebs–Henseleit buffer supplemented with BSA (6 g/100 ml) and human erythrocytes (10% hematocrit). The renal vein was cannulated, and the perfusate flowrate was allowed to stabilize for about 15 min. Then, increasing concentrations of Bay 41-8543 (10 nM, 100 nM, 1 µM), 10 µM S-Nitroso-N-acetyl-D,L-penicillamine (SNAP, Cayman Chemical, Ann Harbor, USA) and finally 10 nM isoproterenol (positive control, Cayman Chemical, Ann Harbor, USA) were added to the perfusate. Samples of the venous perfusate were collected every 2 min to determine renal blood flow and renin secretion rate, which was calculated as the product of renin activity [determined by enzyme-linked immunosorbent assay (ELISA), see below] and the venous flowrate [ng angI/(h*ml) per g kidney weight]. Renin secretion rate after Bay or SNAP treatment was then conclusively related to renin secretion rate obtained after isoproterenol treatment.

### Quantitative mRNA Analysis

Following salt treatment, the left kidney was excised and halved, stored overnight in a fridge in RNAlater (ThermoScientific, Braunschweig, Germany) for penetration into tissue, and then transferred into −80°C until further processing. Total RNA was isolated from the kidney using the PeqGOLD TriFast^™^ reagent (PeqLab, Erlangen, Germany) for phenol/chloroform extraction. After determination of concentration and quality control, 2 µg RNA was reverse transcribed (0.5 µg oligo-dT-Primer, 200 U M-MLV-RT, 20 U RNAsin) and 2 µl cDNA were assayed for renin (forward: 5’-ATGAAGGGGGTGTCTGTGGGGTC-3’, reverse: 5’-ATGCGGGGAGGGTGGGCACCTG-3’) and GAPDH as housekeeper (forward: 5’-CACCAGGGCTGCCATTTGCA-3’, reverse: 5’-GCTCCACCCTTCAAGTGG-3’) using the Roche LightCycler 480 system with SYBR green I (Roche, Mannheim, Germany) as fluorescent dye. For relative quantification, the ∆∆Ct method was used.

### Western Blot Analysis

Proteins were extracted from whole kidneys in 2% lubrol (20 mM Tris, 140 mM NaCl, 2% nonaethylenglycolmonodecylether, 1 mM benzamidine, 0.5 µg/µl leupeptin, 0.3 mM PMSF, PhosStop, pH = 8.0) using an ultraturrax, and 50–70 µg of total protein was loaded on each lane. Following 11.5–12% SDS-PAGE, proteins were blotted on a polyvinylidine fluoride (PVDF) membrane and incubated with renin antibody (R&D systems, Wiesbaden-Nordenstadt, Germany). Vinculin (R&D systems, Wiesbaden-Nordenstadt, Germany) served as loading control. Secondary antibodies were purchased from Dianova (Hamburg, Germany), and bands were detected with the ChemiDoc system (Bio-Rad, Munich, Germany). Quantification was performed using the ImageLab software (Bio-Rad, Munich, Germany).

### Immunohistochemistry

Four-micrometer slices of perfusion-fixed (3% para-formaldehyde) paraffin embedded kidneys were stained for PKGI (Geiselhöringer et al., 2004), renin [either chicken anti-renin (Machura et al., 2009) or goat anti-renin (R&D systems, Wiesbaden-Nordenstadt, Germany)] or α-smooth muscle actin (α-SMA; Beckman Coulter, Germany). Secondary fluorophore-coupled antibodies were obtained from Dianova, Hamburg, Germany. Fluorescence was detected using an Axiovert 200 microscope (objective: 20x; Zeiss, Jena, Germany) and analyzed using the axiovision software. Kidneys from at least three different animals were stained per treatment, whereof one representative staining is shown.

### Plasma Renin Concentration

For the determination of renin concentration in plasma by ELISA (Angiotensin I (PRA) ELISA; DB52011: IBL International, Germany), blood was drawn either from the fascial vein or from the retrobulbar plexus under isoflurane anesthesia. Following centrifugation (2,000×*g*, 20 min), plasma was stored at −80°C until further processing. Alternatively, venous perfusate obtained from isolated perfused kidneys was centrifuged at 1,500×*g* for 10 min and stored at –20°C. For the determination of renin concentration, plasma was diluted 1:10–1:1,000 (depending on treatment) with maleic acid buffer (33.5 mM TRIS, 50 mM maleic acid, 10 mM EDTA; pH = 6.0) to a final volume of 100 µl and 1 µl PMSF (kit content), 40 µl renin substrate (= plasma from bilaterally nephrectomized male rats), as well as 15 µl generation buffer (kit content) was added. Samples were then divided into three fractions, whereas one part was incubated at 4°C for 90 min and the other two parts as duplicate at 37°C for 90 min. Continuation followed according to the manufacturer. Calculation of results was also performed following manufacturer’s recommendation: (value mean − blank value) × 0.74.

### Statistical Analysis

All data are expressed as mean ± SEM, whereas *N*, as indicated in figures, represents biological replicates. The sample size was determined using the G-power 3.1 program. Calculation of statistical differences was performed using GraphPad Prism 5. For the calculation of significant differences between different means, one-way analysis of variance (ANOVA) followed by Bonferroni posttest was performed. If unequal variances were calculated, Kruskal–Wallis test was performed followed by Dunn’s posttest. The levels of significance (*p* values) were considered as follows: <0.05 significant, <0.01 and <0.001 highly significant and stated as *, **, and *** in figures.

## Results

### PKGI Is Expressed in the Juxtaglomerular Apparatus and Can be Deleted Selectively

**Figure 1:**
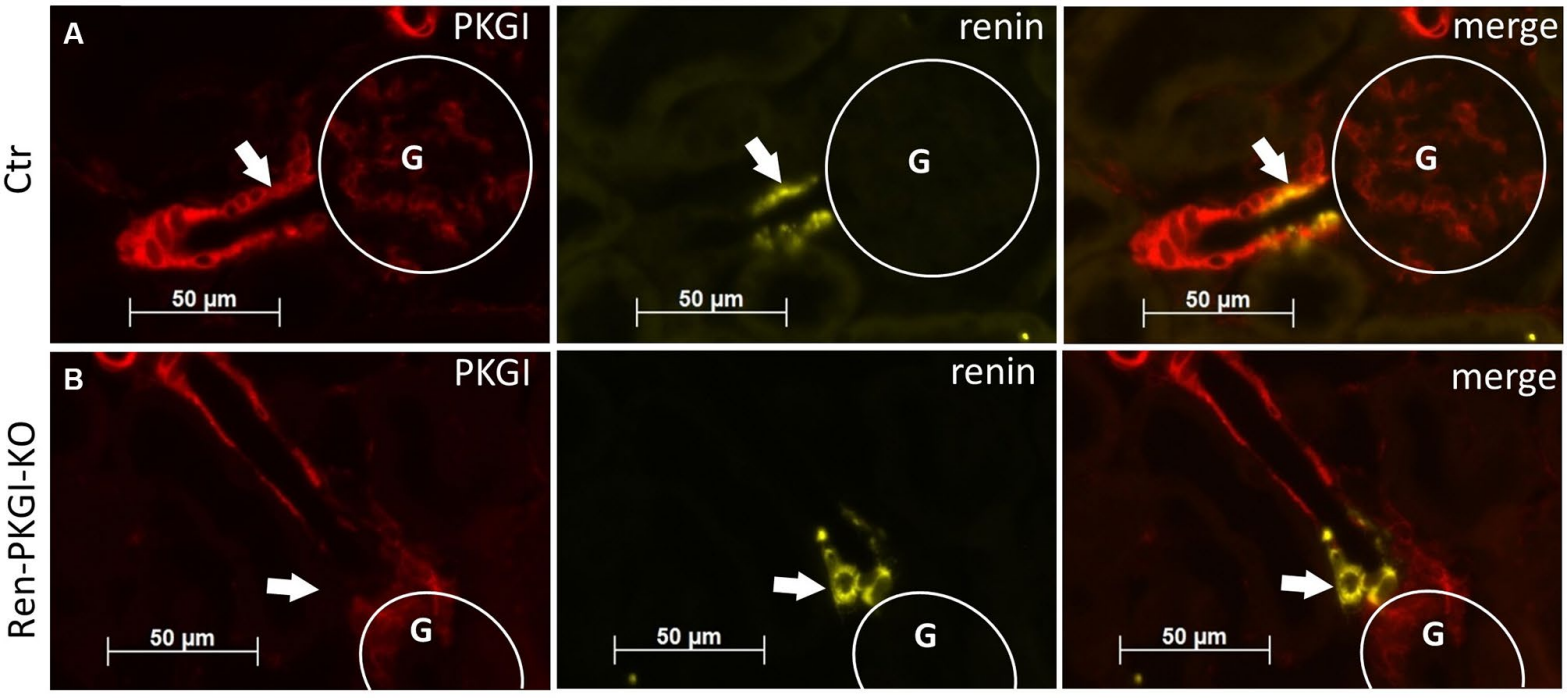
Validation of knockout strategy: Immunohistochemical costainings from kidneys of **(A)** Ctr and **(B)** Ren-PKGI-KO animals. Red: PKGI; yellow: renin. Arrows indicate either **(A)** costaining or **(B)** absent expression in the JGA. G, glomerulus.

First, we validated whether our conditional knockout strategy worked. Therefore, we used kidneys from Ctr as well as Ren-PKGI-KO animals and costained them with renin and PKGI (**Figure 1**). We clearly detected a costaining of PKGI and renin in Ctr kidneys and therefore an expression of this kinase in JG cells (**Figure 1A**, arrows indicate costaining), confirming our previous results (Schinner et al., 2013). In contrast, in mice lacking PKGI in the JG cells due to an expression of Cre recombinase under the Renin1d promotor, we did not observe any colocalization of PKGI and renin anymore (**Figure 1B**, arrows indicate absent costaining), whereas expression of PKGI in arterioles was not influenced. Hence, we conclude that Ren-PKGI-KO animals are a suitable model for studying PKGI actions in JG cells.

### PKGI Is Not Chiefly Involved in the Chronic Regulation of Renin Synthesis, Secretion, or Recruitment Following Different Salt Diets

Next, we challenged the RAAS in these mice *via* stimulation (low salt, LS; low salt + enalapril, LS/Ena) or inhibition (high salt, HS) of renin synthesis. At first, we measured systolic blood pressure (SBP) in conscious animals *via* tail cuff plethysmography (**Supplemental Table 1**). We did not detect any differences during a normal salt diet between genotypes. Following 3 weeks of LS/Ena treatment, SBP of control animals was significantly lowered to 75% of the base value, as expected. However, SBP of Ren-PKGI-KO mice also dropped but interestingly not as pronounced as in the controls (87% of base value, significant difference vs. control).

**Figure 2:**
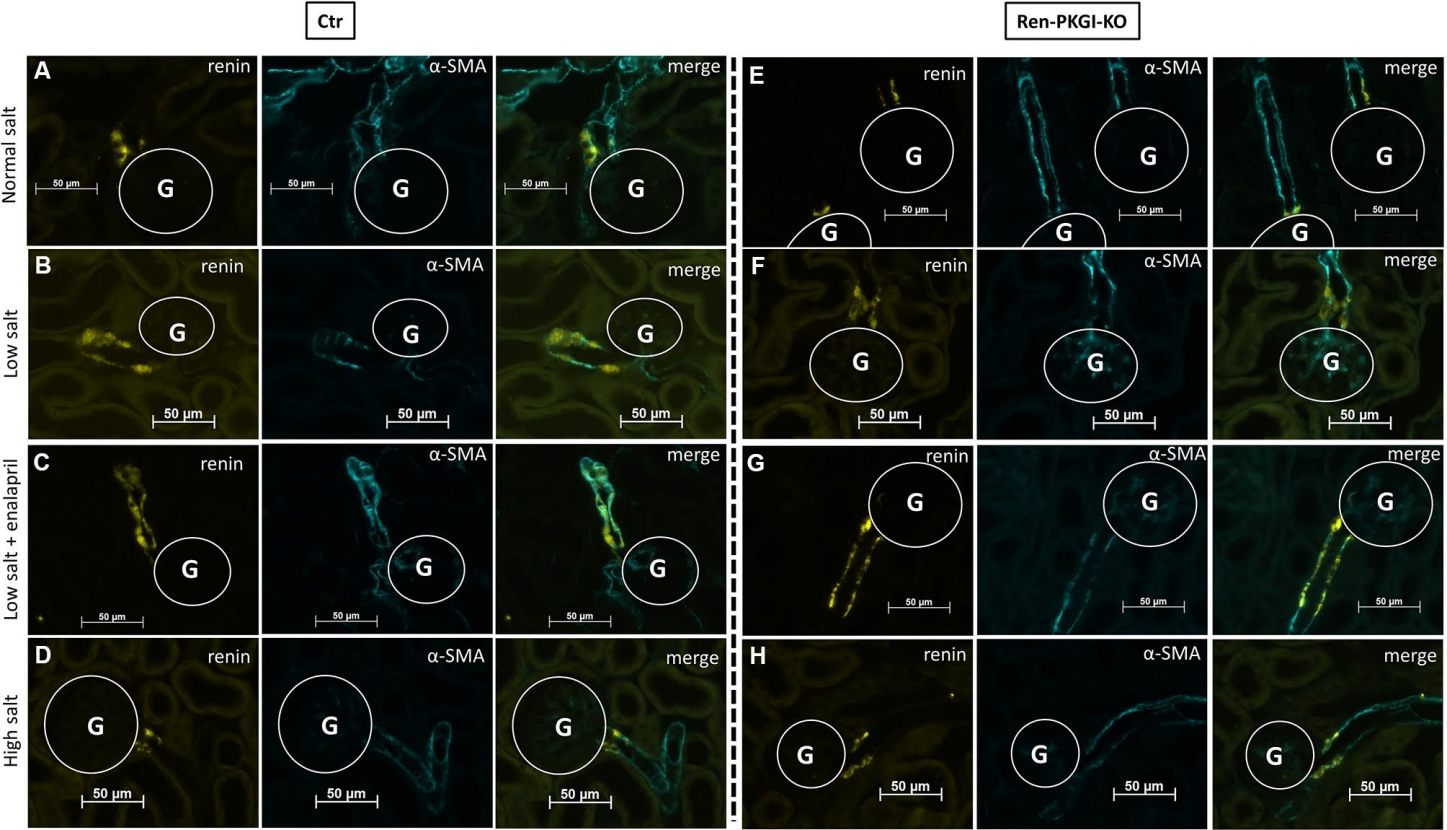
Immunohistochemical analysis of the juxtaglomerular apparatus (JGA) during different salt loads: Representative costainings of renin (yellow) and α-smooth muscle actin (α-SMA, turquoise). **(A–D)** Ctr; **(E–H)** Ren-PKGI-KO. **(A, E)** normal salt (NS); **(B, F)** low salt (LS); **(C, G)** low salt + enalapril (LS/Ena); **(D, H)** high salt (HS). Stainings were repeated at least three times from different animals. G, glomerulus.

Immunohistochemistry (IHC) images of Ctr animals (**Figure 2A–D**) showed a clear upregulation of renin expression following LS treatment, which was even more pronounced in LS/Ena-treated mice, concordant with the abolished negative feedback control of AngII upon angiotensin conversion enzyme (ACE) blockade. Inhibition of renin synthesis by HS resulted in a slight downregulation of renin. In Ren-PKGI-KO animals (**Figure 2E–H**), apparently, the same changes in renin expression were detected. Regarding renin recruitment following LS/Ena treatment, no differences occurred between the two genotypes. We noticed a transformation of preglomerular vascular smooth muscle cells to renin-producing cells (**Figure 2C and G compared to A and E**) and hypertrophy of juxtaglomerular cells, resulting in the so-called cuff-like structures around the glomerulus in both genotypes (**Supplemental Figure 1**).

**Figure 3:**
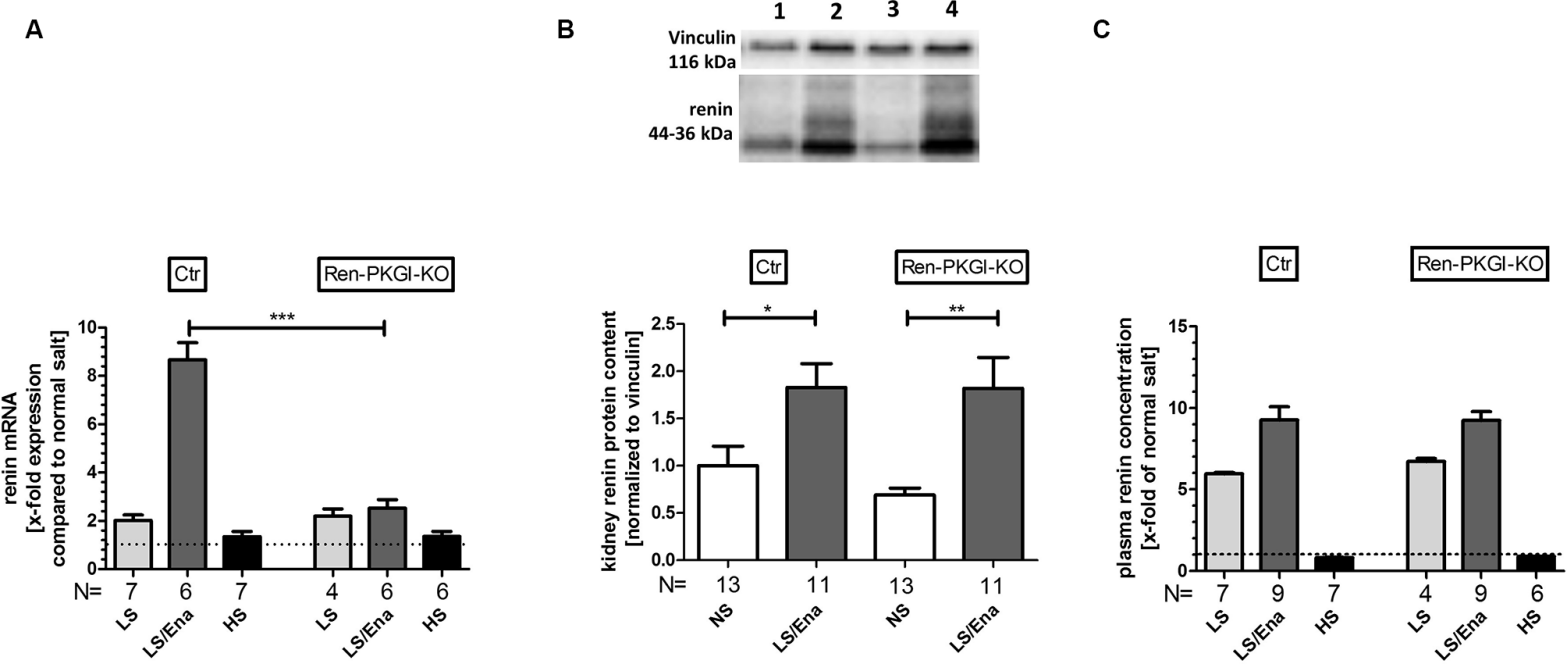
Renin mRNA, renin protein and plasma renin concentration (PRC) during different salt loads: **(A)** renal renin mRNA, relative quantification calculated per ΔΔCt compared to normal salt treated animals of the same genotype. **(B)** representative Western blot and relative quantification concerning renal renin protein content. Western blot was loaded with 70 µg protein/lane and incubated with anti-vinculin (116 kDa) as loading control and anti-renin (44–36 kDa). Lane 1: Ctr, normal salt; lane 2: Ctr, low salt + enalapril; lane 3: Ren-PKGI-KO, normal salt; lane 4: Ren-PKGI-KO, low salt + enalapril. Statistical analysis shows relative expression of renin normalized to vinculin following LS/Ena compared to normal salt treated animals of the same genotype. *^,^**: (highly) significant difference (Kruskal–Wallis test). **(C)** relative PRC compared to normal salt treated animals. ***: highly significant difference between LS/Ena-treated mice of different genotypes (ANOVA, *p* < 0.001).

To quantify our observations, we performed mRNA and protein analysis from kidney tissue and determined PRC. Regarding renin mRNA (**Figure 3A**), an about twofold upregulation was detected in both genotypes following LS treatment. Under additional ACE inhibition, control animals further enhanced renin-mRNA generation about ninefold to NS treatment, this upregulation was abolished in mice lacking PKGI in JG cells. Following HS treatment, both genotypes had renal renin mRNA levels comparable to NS-treated animals.

Since mRNA levels of renin were highly significantly altered after LS/Ena treatment, we were also interested if this difference was also reflected in renal renin protein content. However, both genotypes responded similarly with an about twofold upregulation of protein upon LS/Ena stimulation as assessed *via* quantitative Western blot (**Figure 3B**). Moreover, renin protein amounts following LS and HS treatments also did not differ between both genotypes as already implied by mRNA levels (data not shown). Considering that an altered electrolyte handling induced by different salt diets might also cause an adapted renal expression of the PKGs, we estimated the protein concentrations of PKGIα and PKGII *via* Western blot (**Supplemental Figure 2**). We did not detect an obvious change in expression of neither PKG isoform indicating that, at least in the murine kidney, different salt loads do not lead to a modified protein concentration of PKG.

We also analyzed PRC (**Figure 3C**) *via* ELISA. By LS treatment, PRC was enhanced in both genotypes to a similar extent compared to NS. Additional treatment with enalapril further exceeded PRC in both genotypes, comparable to the enhancement seen in mRNA levels. Inhibition of the RAAS by high salt diminished PRC between 10% (Ren-Cre/PKGI fl/fl) and 20% (PKGI fl/fl) compared to normal salt conditions.

### Knockout of Both Expressed PKG Isoforms Also Does Not Influence Chronic Renin Regulation

**Figure 4:**
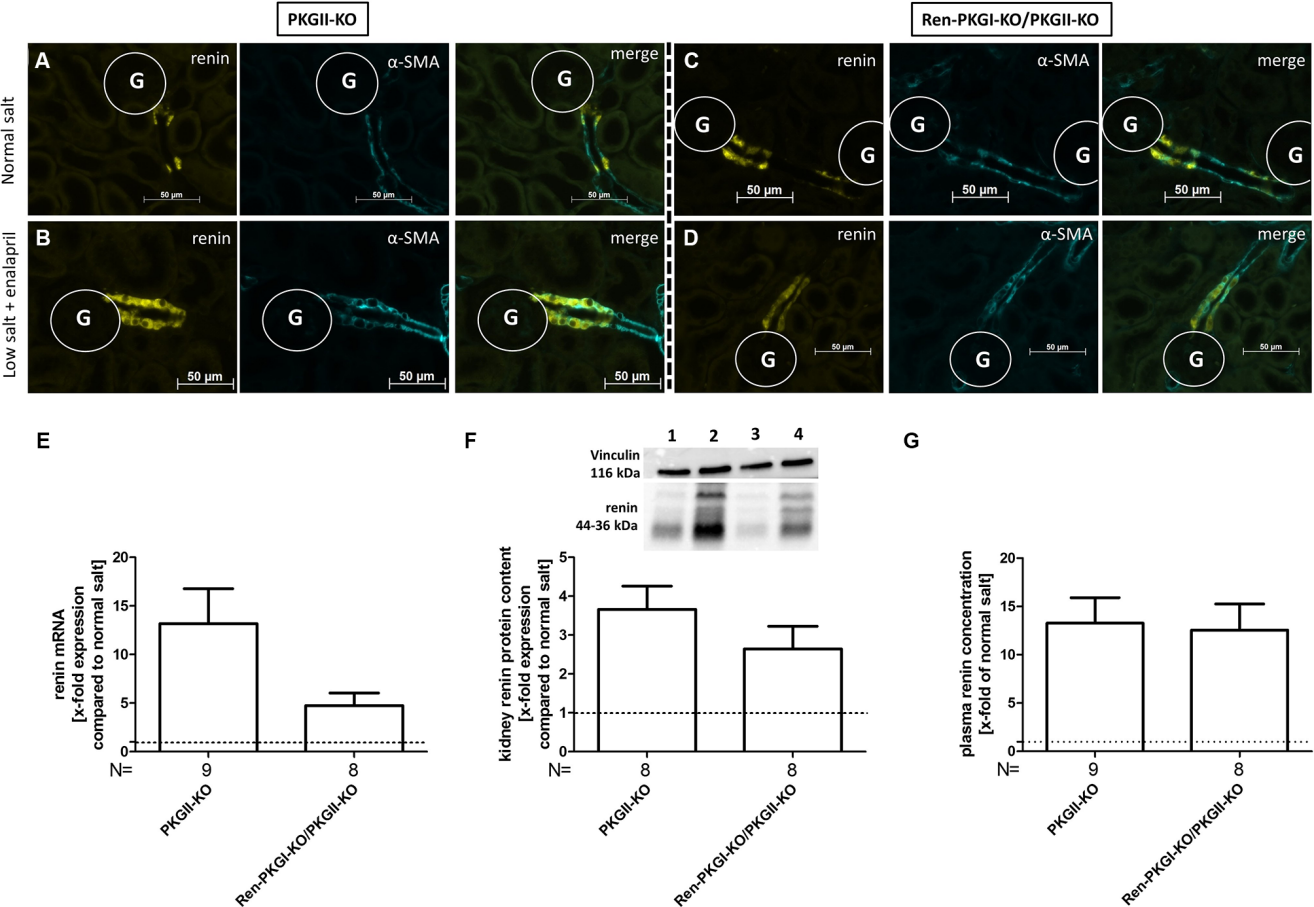
Renin analysis of double knockouts during NS and LS/Ena treatment: **(A–D)** representative costainings of renin (yellow) and α-smooth muscle actin (α-SMA, turquoise). **(A**, **B)** PKGII-KO; **(C**, **D)** Ren-PKGI-KO/PKGII-KO. **(A**, **C)** normal salt; **(B**, **D)** low salt + enalapril; stainings were repeated at least three times from different animals. G, glomerulus. **(E)** renal renin mRNA, relative quantification of LS/Ena treatment calculated per ΔΔCt compared to normal salt treated animals of the same genotype. **(F)** representative Western blot and relative quantification concerning renal renin protein content. Western blot was loaded with 70 µg protein/lane and incubated with anti-vinculin (116 kDa) as loading control and anti-renin (44–36 kDa). Lane 1: PKGII-KO, normal salt; lane 2: PKGII-KO, low salt + enalapril; lane 3: Ren-PKGI-KO/PKGII-KO, normal salt; lane 4: Ren-PKGI-KO/PKGII-KO, low salt + enalapril. Statistical analysis shows relative expression of renin upon LS/Ena treatment compared to normal salt treated animals of the same genotype. **(G)** relative plasma renin concentration compared to normal salt treated animals.

Since both PKGIα and PKGII are expressed in JG cells (Gambaryan et al., 1996; Wagner et al., 1998; Schinner et al., 2013) we considered, if a lack of PKGI was compensated by PKGII, therefore, an involvement of PKGI in renin regulation could be masked by the presence of the other kinase. Hence, we mated the aforementioned mice with mice carrying a PKGII null mutation (PKGII-KO) and reanalyzed these animals by applying NS and LS/Ena diets, whereby animals which did not contain the Cre recombinase (PKGII-KO) served as controls (**Figure 4A–G**). Again, we observed an upregulation of renin mRNA following LS/Ena treatment (**Figure 4E**) especially in mice where only PKGII was deleted (PKGII-KO). In double-knockout animals (Ren-PKGI-KOPKGII-KO), the increase in renin mRNA was not as pronounced as in single PKGII-KOs without reaching statistical significance. This trend was continued in protein levels, since double KOs tended to have a somewhat lower renin protein concentration following LS/Ena treatment as assessed qualitatively *via* IHC (**Figure 4B and D**) and quantitatively *via* Western blot (**Figure 4F**). However, when analyzing PRC (**Figure 4G**), we detected an LS/Ena-dependent upregulation in single as well as in double Kos, while the aforementioned differences were not observed anymore.

### Prolonged Stimulation of Soluble Guanylate Cyclase Leads to Recruitment of Preglomerular Renin Cell Precursors to Renin-Expressing Cells Independently of PKG

**Figure 5:**
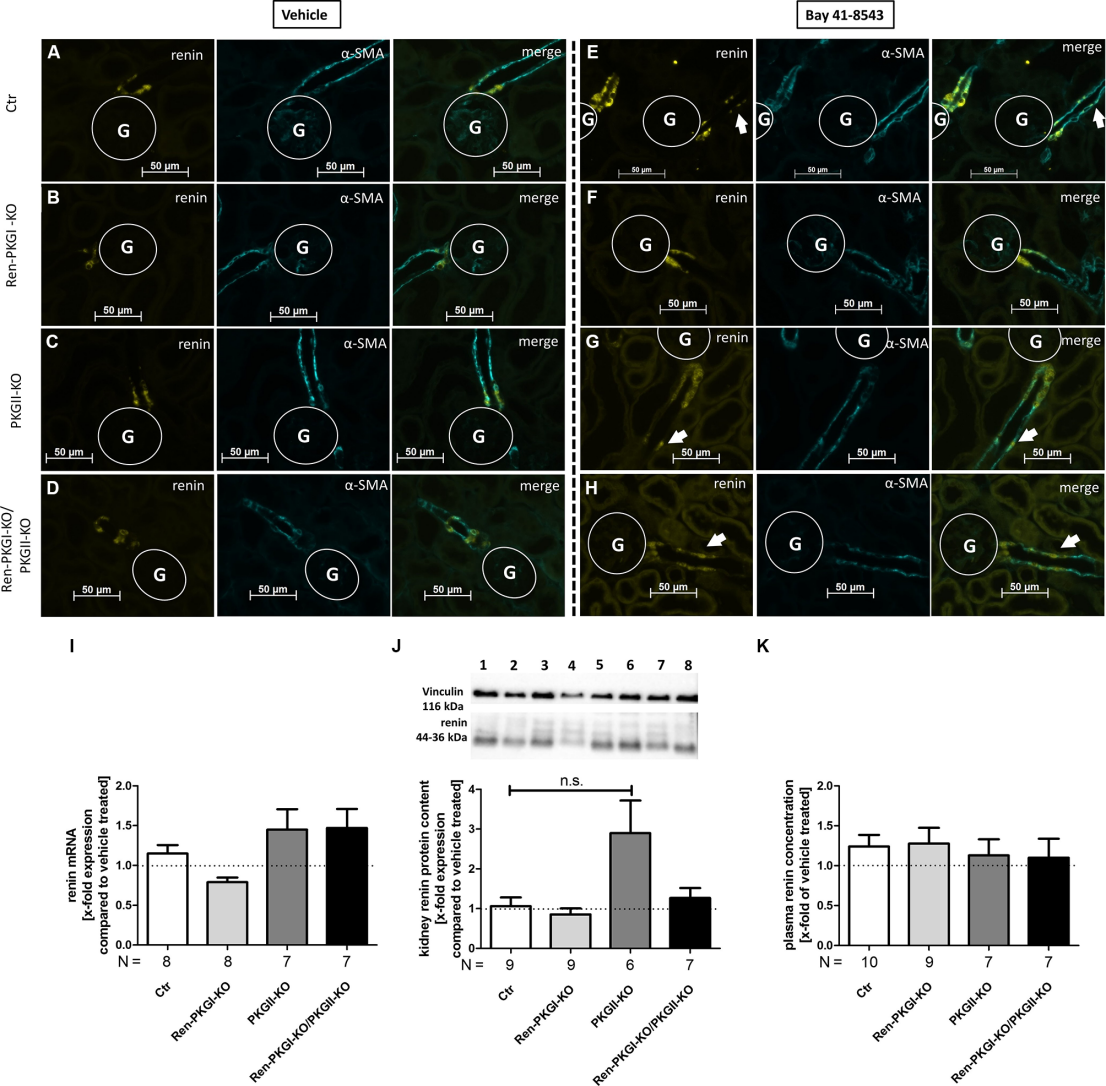
Renin analysis after chronic soluble guanylate cyclase (sGC) stimulation: Mice were treated either with vehicle or with Bay 41-8543 (1 mg/kg BW) *via* intraperitoneal injection once a day for 1 week. **(A–H)** representative costainings of renin (yellow) and α-smooth muscle actin (α-SMA, turquoise); vehicle-treated mice: **(A–D)** Bay-treated mice; **(E–H)** **(A, E)** Ctr; **(B, F)** Ren-PKGI-KO; **(C, G)** PKGII-KO; **(D, H)** Ren-PKGI-KO/PKGII-KO. Stainings were repeated at least three times from different animals. Arrows indicate recruited renin-expressing cells following Bay treatment. G, glomerulus. **(I)** renal renin mRNA, relative quantification calculated per ΔΔCt compared to vehicle-treated animals of the same genotype (Kruskal–Wallis test). **(J)** representative Western blot and relative quantification concerning renal renin protein content. Western blot was loaded with 70 µg protein/lane and incubated with anti-vinculin (116 kDa) as loading control and anti-renin (44–36 kDa). Lane 1: Ctr, vehicle-treated; lane 2: Ctr, Bay-treated; lane 3: Ren-PKGI-KO, vehicle-treated; lane 4: Ren-PKGI-KO, Bay-treated; lane 5: PKGII-KO, vehicle-treated; lane 6: PKGII-KO, Bay-treated; lane 7: Ren-PKGI -KO/PKGII-KO, vehicle-treated; lane 8: Ren-PKGI-KO/PKGII-KO, Bay-treated. Statistical analysis shows relative expression of renin compared to vehicle-treated animals of the same genotype (Kruskal–Wallis test). **(K)** relative plasma renin concentration compared to vehicle-treated animals of the same genotype.

As a previous study demonstrated that sGC is responsible for renin recruitment during LS/Ena treatment, we wondered if we could cause recruitment in normal salt treated animals by applying the sGC stimulator Bay 41-8543. At first, we confirmed the expression of sGC (subunit β1) in juxtaglomerular cells of all genotypes by IHC stainings costained with renin (**Supplemental Figure 3A–D**). We also validated whether the genetic knockout of PKGs in these animals itself leads to an altered sGC regulation without any further manipulation (**Supplemental Figure 3E, F**). By analyzing mRNA and protein contents of whole kidney homogenates, we did not detect any difference in the expression levels of sGCβ1 between the different genotypes, suggesting that sGC expression is not altered when one or both PKG isoforms are absent. Since it is known that Bay 41-8543 can decrease blood pressure significantly (Stasch et al., 2002), we assured that the applied dose did not induce a decrease in blood pressure (**Supplemental Table 1**). SBP remained stable before and after 1 week of a once daily i.p. treatment with 1 mg/kg Bay 41-8543, suggesting that possible changes in renin regulation after this time period are not a consequence of altered blood pressure but of a direct effect. We then treated animals either with vehicle or with Bay 41-8543 (1 week, 1 mg/kg i.p., once daily) and analyzed renin recruitment by costainings of renin and α-SMA (**Figure 5A–H**). Upon sGC stimulation, we observed occasionally occurring renin-positive cells located distally from the juxtaglomerular apparatus (JGA), confirming the suggested role of sGC for renin recruitment (**Figure 5E–H**, arrows indicate renin-positive cells), although the majority of JG cells showed a staining similar to vehicle-treated animals (an example of a nonrecruited JGA for each genotype is shown in **Supplemental Figure 4**). This effect was not mediated by PKGs, as all genotypes responded similarly. Neither renin mRNA nor overall kidney renin protein content was altered following prolonged sGC stimulation compared to vehicle-treated animals in any genotype (**Figure 5I, J**), which also was reflected by unchanged PRC levels (**Figure 5K**). Since administration of drugs can lead to altered expression levels of the targeted protein, we examined renal sGC-mRNA and protein content (**supplemental Figure 5A, B**). We did not observe any differences in neither genotype of Bay 41-8543-treated animals compared to vehicle-treated animals, indicating that Bay 41-8543 did not influence transcriptional or translational processes of sGC. We also controlled systolic blood pressure before and 7 days after Bay 41-8543 treatment in Ctr and Ren-PKGI-KO mice (**Supplemental Table 1**). However, we did not detect any differences, which suggests that 1 mg/kg of the sGC stimulator does not induce any lasting decrease in blood pressure.

### Acute Stimulation of Soluble Guanylate Cyclase Leads to an Enhanced Renin Secretion Which is Abolished When PKG is Missing

**Figure 6:**
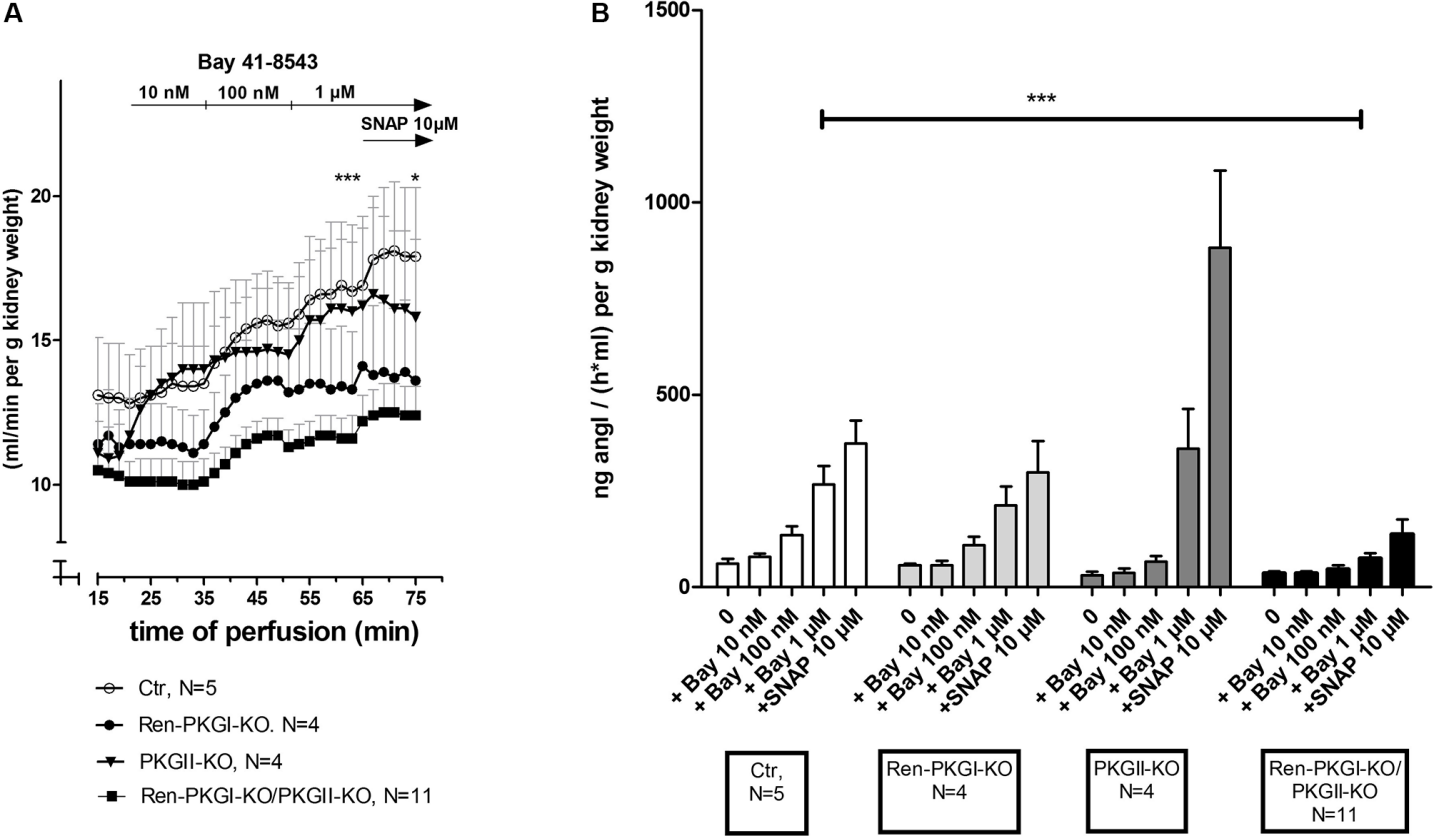
Effect of sGC-stimulation on renin secretion rate in the isolated perfused kidney: Isolated perfused kidneys of either genotype were treated with increasing concentrations of Bay 41-8543 (10 nM–1 µM) followed by the NO-donor SNAP (10 µM) as positive control. **(A)** Perfusate flow of isolated perfused kidneys of Ctr (control, white circle), Ren-PKGI-KO (black circle), PKGII-KO (black triangle), or Ren-PKGI-KO/PKGII-KO (black square). Data are presented as mean ± SEM of all animals of the same genotype at each time point. *^,^***: (highly) significant difference between 1 µM Bay- or SNAP-treated kidneys of Ctr vs. Ren-PKGI-KO/PKGII-KO (*t* test, *p* < 0.05 for *, *p* < 0.001 for ***). **(B)** Renin secretion rate of isolated perfused kidneys of Ctr (white), Ren-PKGI-KO (light gray), PKGII-KO (dark gray), or Ren-PKGI-KO/PKGII-KO (black). ***: highly significant difference between Bay-treated kidneys of Ctr vs. Ren-PKGI-KO/PKGII-KO as indicated (ANOVA, *p* < 0.001).

We were not only interested in chronic effects of Bay 41-8543 but also if an acute treatment altered renin regulation. Initially, we used the model of the isolated perfused kidney (IPN), which enables the measurement of perfusate flow and secreted renin directly after the perfusate passed the kidney. We analyzed both parameters in kidneys of all genotypes after adding increasing concentrations of Bay 41-8543 to the perfusate (10 nM, 100 nM, 1 µM). Regarding perfusate flow (**Figure 6A**), we ended up by adding 10 µM of the NO-donor SNAP (S-Nitroso-N-acetyl-D,L-penicillamine; positive control). In kidneys of control animals (Ctr), we detected an increase in perfusate flow following increasing Bay 41-8543 concentrations, which could be slightly enhanced by the final addition of SNAP. In contrast, in kidneys from mice lacking either one or both PKG isoforms, this effect was less pronounced with the strongest (and statistically significant) difference in kidneys of double-knockout mice [18% increase, compared to control (37% increase)]. To study and evaluate the efficiency of an sGC stimulation on renin secretion, we studied the renin concentration found in the perfusate following Bay 41-8543 and SNAP treatment (**Figure 6B**). While 10 nM Bay 41-8543 had no effect on renin release rate, 100 nM Bay 41-8543 induced a moderate renin secretion, and 1 µM Bay 41-8543 caused a marked increase (approximately fourfold of nontreated) in kidneys of control animals (Ctr, white bars). The final stimulation with SNAP led to a slightly further enhanced renin release. When we analyzed kidneys of Ren-PKGI-KO animals (light gray bars), we detected quite similar increases in renin release compared to control animals. Kidneys of PKGII-KO (dark gray bars) also reacted with a clear increase in renin release upon Bay stimulation. However, renin release upon SNAP infusion was even more prominent (more than doubled) in kidneys of these animals compared to SNAP-infused kidneys of control animals. In contrast, kidneys of double-knockout animals (Ren-PKGI-KO/PKGII-KO, black bars) did not respond at all on Bay 41-8543, and the impact of SNAP was substantially diminished compared to control animals. Since we could not be sure if these kidneys in general reacted with a reduced renin release, we finally also infused isoproterenol, an inductor known to cause a strong renin release. However, both kidneys of control animals and kidneys of double-knockout animals increased renin release similarly in response to isoproterenol stimulation [713 vs. 900 ng angI/(h ml) per g kidney weight, data not shown].

**Figure 7:**
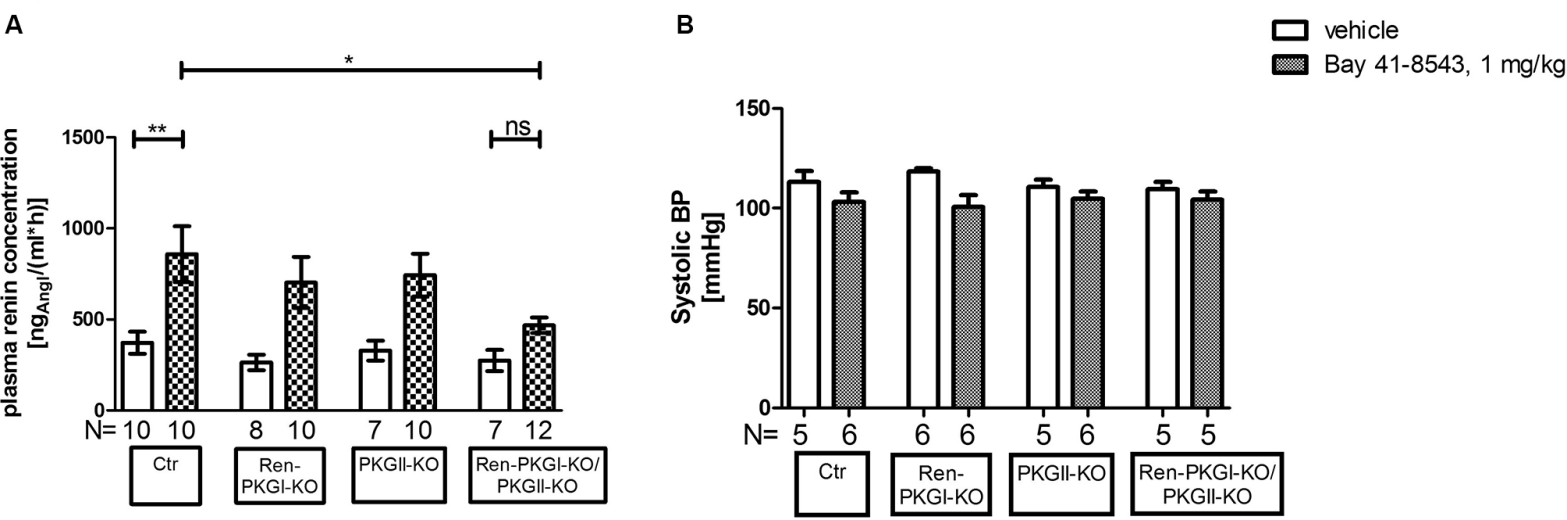
Renin analysis 45 min after sGC stimulation: Mice were treated either with vehicle or with Bay 41-8543 (1 mg/kg BW) *via* intraperitoneal injection for 45 min. Data are presented as mean ± SEM. **(A)** plasma renin concentration of vehicle vs. Bay-treated animals of different genotypes. *^,^**: (highly) significant difference either between Ctr vehicle-treated vs. Bay-treated or between Ctr Bay-treated vs. Ren-PKGI-KO/PKGII-KO Bay-treated (ANOVA, *p* < 0.05 for *, *p* < 0.01 for **). **(B)** systolic blood pressure (SBP) of vehicle (white bars) vs. Bay-treated animals (squared bars). SBP was determined *via* tail cuff plethysmography, whereas mice were trained for three to four consecutive days prior to measurement. Three to ten measurements/animal were averaged.

Since the IPN is an *ex vivo* model, we finally wanted to investigate if these effects were also seen *in vivo*. Therefore, we applied Bay 41-8543 i.p. to animals of all genotypes and determined the PRC 45 min postinjection (**Figure 7A**). In control animals as well as in both single knockouts, the PRC was more than doubled compared to that in vehicle-treated animals of the same genotype. However, double-knockout animals almost completely failed to respond to the Bay injection; we detected only a slight and nonsignificant increase in PRC compared to vehicle-treated mice. In contrast, the difference upon Bay stimulation between Ctr control animals and Ren-PKGI-KO/PKGII-KO double knockouts reached statistical significance (*p* < 0.05), hence reflecting the previously obtained results in the IPN experiments.

Considering that blood pressure is a crucial regulator of renin secretion, we supposed that a varying blood pressure response to Bay 41-8543 in the four genotypes might account for the differences seen in PRC. Hence, we analyzed systolic blood pressure (SBP) after an acute treatment with Bay 41-8543 compared to vehicle treatment (**Figure 7B**). As expected, SBP dropped slightly, however nonsignificantly following sGC stimulation in accordance to the vasodilating effect of cGMP. Importantly, this effect was observed in all genotypes, indicating that blood pressure differences were not responsible for the effects on PRCs. Moreover, sGC expression was not altered by the genetic manipulations in the different genotypes (**Supplemental Figure 3**), excluding that different levels of sGC led to a varying response to Bay 41-8543. Therefore, we conclude that the acute effects seen on PRC are dependent of PKGs.

## Discussion

In this study we have examined renin regulation by cGMP/PKG. For these studies, we used single- and double-knockout mice of renin-cell-specific PKGI-KO and of total PKGII-KO under basal and renin–angiotensin–aldosterone system (RAAS)-stimulating and -inhibiting conditions. Furthermore, we studied cGMP effects upon sGC stimulation in these animals and in isolated perfused kidneys (IPN). Thereby, we have shown that cGMP/PKG does not alter chronic renin balance regarding renin release and recruitment but is involved in acute changes of renin secretion.

As stated above, basal renin regulation and chronic stimulation of the RAAS was not changed upon PKG deletion. An obvious explanation is that, under these chronic conditions, NO/cGMP-mediated effects on renin secretion and recruitment are not dependent on PKGs. This is in accordance to several previous reports which state that renin regulation is chiefly controlled by the cAMP/protein kinase A (PKA) system. Importantly, phosphodiesterases (PDEs) allow a crosstalk between the cGMP and the cAMP system. NO/cGMP-mediated regulation of renin responses is thought to be mainly controlled by PDE3 (Schweda and Kurtz, 2004). This PDE isoform is inhibited by cGMP but has a substrate specificity for both cGMP and cAMP, thus enabling an increase in cAMP following cGMP binding and subsequent PKA activation. Several previous reports stated that cGMP crosstalk *via* PDE3-mediated cAMP rise is responsible for the stimulation of renin secretion (summarized in Szaszák et al., 2008). Another explanation could be that counter regulations regarding adrenal release of aldosterone might mask cGMP-specific effects. PKGI and PKGII were found to be expressed in the adrenal gland. Previous studies have shown that PKGII is expressed in the zona glomerulosa. It was reported that basal aldosterone release is involved by PKGII in isolated rat zona glomerulosa cells (Gambaryan et al., 2003). However, murine PKGII deletion did not change aldosterone release under basal conditions and upon LS treatment (Spiessberger et al., 2009). The role of PKGI in the adrenal gland is uncertain. Ren1d expression was previously found in fetal adrenal gland (Fabian et al., 1989), but its expression was not revealed in the adult adrenal gland. Therefore, we performed IHC stainings to validate whether PKGI is deleted in the adrenal gland of the Ren-PKGI-KO mice (data not shown). We did not detect a deletion of PKGI but could endorse the previous studies by Gambaryan et al. that PKGI is only expressed in the capsule and blood vessels but not found in the zona glomerulosa (Gambaryan et al., 2003). Moreover, aldosterone serum concentrations were not altered between Ctr and Ren-PKGI-KO animals, at least under control conditions (data not shown). Hence, it can be assumed that counter regulations by aldosterone did not mask any effects on renin regulation. Furthermore, the acute changes in IPN argue for kidney-specific effects of PKGs. It should be noted that we observed significant differences regarding renin mRNA level following LS/Ena treatment, preferentially in animals, where PKGI was missing in the JG cell (Ren-PKGI-KO and Ren-PKGI-KO/PKG II-KO, **Figures 3A** and **4E**). Some recent studies suggest that PKGs might affect transcriptions of different genes by phosphorylating transcription factors (Lee et al., 2015; Ma et al., 2016). We can only speculate that these mechanisms might also lead to a diminished renin transcription. However, since renal renin protein as well as PRCs remained unaltered compared to control animals, this finding seems to be of minor importance in terms of overall renin regulation.

Contradictory data have been published regarding the impact of the cGMP system on renin regulation (Hofmann et al., 2006). Most reports support the view that cGMP decreases RAAS activity. Previous reports suggested that cGMP-activated PKGII and possibly PKGI inhibit renin secretion (Gambaryan et al., 1998; Wagner et al., 1998). However, these experiments were performed mainly in isolated juxtaglomerular cells. Therefore, it might be that, upon culturing, these cells altered their differentiation status so that these conditions are not comparable to other studies.

Our results confirm that positioning of renin-secreting cells upon enalapril/low salt treatment is dependent on NO-GC (Neubauer et al., 2013). Interestingly, renin recruitment was not changed upon deletion of PKGI and II. Hence, these results indicate that renin recruitment under chronic conditions is dependent on cGMP, but is PKG independent.

Our results indicate that PKG enzymes are involved in acute renin secretion. In our experiments of IPN and of acutely Bay-treated animals, renin release, which was enhanced by Bay application, was strongly affected by the deletion of both kinases. These results suggest that stimulation of renin secretion by cGMP is mediated by PKGI and II. Interestingly, PKGII deletion alone had a stronger effect on renin secretion than single renin-cell-specific deletion of PKGI. Therefore, it can be concluded that PKGII is more strongly involved in this stimulatory effect of BAY on renin release than PKGI. In contrast, application of SNAP lead to a strong stimulatory effect in PKGII-KO mice This indicates that, at high NO concentrations, PKGII might exhibit inhibitory effects on renin secretion. Neubauer et al. previously have shown using Ren1d+/Cre–NO-GCfl/fl kidneys that application of the NO-donor sodium nitroprusside (SNP) enhanced perfusate flow and that this effect was dependent on the presence of NO-GC in renin-expressing cells (Neubauer et al., 2013). Previously, it was shown that the application of NO donors enhanced perfusate flow of isolated rat kidney. It was suggested that cGMP inhibition of PDE3 mediated these effects (Kurtz et al., 1998). In our studies, the application of the sGC stimulator Bay-41-8543 also enhanced the perfusate flow. Deletion of total PKGII or renin-cell-specific PKGI alone did not significantly alter Bay-mediated stimulation of perfusate flow. However, this outcome was absent in the double knockout, suggesting that both PKG enzymes, PKGI and II, are involved in perfusate flow. Hence, the effect of the double-knockout indicates that PKG enzymes compensate each other regarding renin secretion. An altered expression of sGC in these animals was not found and therefore cannot be the reason for this effect. It might be argued that, in these mice, a vasodilation which is normally induced by sGC-generated cGMP is missing. This could be responsible for the observations on perfusate flow, as expression of the Renin1d promotor using Renin1d-GFP reporter was previously shown in isolated microvessels (Pentz et al., 2001). Furthermore, in Ren1d+/Cre–NO-GCfl/fl mice, the NO-GC protein vanished in preglomerular smooth muscle cells in line with studies which demonstrated that preglomerular smooth muscle cells are derived from renin-expressing cells (Neubauer et al., 2013). However, our immunohistochemical analysis did not show a deletion of PKGI in the preglomerular vessels of the Renin-Cre-specific PKGI knockout. Furthermore, PKGII was previously found in juxtaglomerular cells and in afferent arterioles (Gambaryan et al., 1996). Therefore, altered perfusate flow in the double knockout cannot be excluded by a reduced dilative response upon PKGII deletion. However, this is unlikely as no effect on perfusate flow was observed in the single total PKGII-KO. Furthermore, there is no evidence that PKGII is involved in vascular smooth muscle contractility, and there were no effects of PKGII deletion on blood pressure observed. Additionally, blood pressure was nonsignificantly changed by BAY application, excluding that BAY-mediated vasodilation was a main cause for the observed effects on renin secretion.

Others demonstrate that NO/cGMP can also acutely increase PRC. In humans, it has been shown that an inhibition of the inducible NO-synthase (iNOS) by asymmetric dimethylarginine (ADMA) and hence diminished cGMP concentrations leads to a decrease in PRC (Kielstein et al., 2004). However, as renovascular resistance was enhanced simultaneously by the administration of ADMA, the observed decrease in PRC might be a consequence of altered hemodynamics as well. In contrast, a recent study by Chen et al. suggests that acute activation of the particulate guanylyl-cyclase A receptor and hence enhancement of cGMP leads to a decrease in plasma renin activity (Chen et al., 2018). Since these studies were performed in anesthetized dogs, differences regarding the used model organism as well as consciousness of the animals have to be taken into consideration.

Next, it would be interesting to elucidate which mechanisms and substrates of PKGI and/or PKGII are mediating the acute stimulation of renin secretion. In several organs, it was shown that PKGI reduces intracellular calcium concentrations. Therefore, it might be conceivable that this effect is also found in the renin-secreting cells. Previous studies reported juxtaglomerular cells exhibit the calcium paradoxon, which means that calcium reduction leads to enhanced renin secretion. This mechanism might be also due for the PKGI-mediated renin secretion. Recent results have shown that a mechanism of the calcium paradoxon involves activation of MLCK (Steppan et al., 2018). Therefore, it might be possible that PKGI-mediated renin secretion acts similarly. The mechanism of PKGII can only be speculated. PKGII is membrane bound and involved in secretory processes (Pfeifer et al., 1996; Schramm et al., 2014). In this regard, it might be interesting that cAMP-induced renin release was correlated with SNAP23-mediated exocytosis (Mendez and Gaisano, 2013). Furthermore, the exocytosis of renin-containing granular organelles upon isoproterenol stimulation was shown with high resolution using TIRF microscopy (Buckley et al., 2017). It might be that also cGMP/PKGII enhances exocytotic mechanisms for renin containing granules, and it would be tempting to analyze this process in the future. It remains to be elucidated why chronical and acute effects of cGMP/PKG differ. It can be speculated that compensatory mechanisms, e.g., regulation of PDEs might be the reason for these different responses.

In summary, our study indicates that cGMP/PKG does not induce a change in long-term regulation of renin generation and secretion. Under prolonged stimulation of sGC, renin recruitment is enhanced, an effect independent of PKG. In contrast, when sGC was acutely stimulated, renin secretion was significantly enhanced dependent on the presence of PKG. These results can be relevant during chronic or acute pharmacological application of cGMP-elevating drugs, e.g., PDE5 inhibitors or sGC stimulators (Krishnan et al., 2018).

## Data Availability

The datasets for this manuscript are not publicly available because the raw data supporting the conclusions of this manuscript will be made available by the authors, without undue reservation, to any qualified researcher. Requests to access the datasets should be directed to jens.schlossmann@chemie.uni-regensburg.de.

## Ethics Statement

All animal experiments were performed in accordance with the Guidelines for the Care and Use of Laboratory Animals published by the US National Institutes of Health and approved by the local ethics committee.

## Author Contributions

AS, FS, and JS were involved in the conception, design, and interpretation of the experiments; AS, FS, and JS performed and analyzed the experiments; FH, MS-L, and PS contributed essential material; and AS and JS wrote the manuscript. All authors were involved in the critical revision of the manuscript for important intellectual content.

## Funding

This work was financially supported by the Bavarian State and Sonderforschungsbereich SFB699. MS-L is supported by NIH DK091330, DK096373 and DK116718.

## Conflict of Interest Statement

PS is an employee of the Bayer AG. The remaining authors declare that the research was conducted in the absence of any commercial or financial relationships that could be construed as a potential conflict of interest.
