# Supplementary material for: Protein Kinase G Is Involved in Acute but Not in Long-Term Regulation of Renin Secretion
Source: Front Pharmacol. 2019 Jul 18;10:800. doi: 10.3389/fphar.2019.00800 (PMC6657341; doi:10.3389/fphar.2019.00800)
Supplement: Supplementary file 1 [file Table_1.docx]

Supplementary Material

Protein kinase G is involved in acute but not in long-term regulation of renin secretion

Andrea Schramm^1^, Frank Schweda^2^, Maria Luisa S. Sequeira-Lopez^3^, Franz Hofmann^4^, Peter Sandner^5^ and Jens Schlossmann^1*^

^1^ Institute of Pharmacy, Department of Pharmacology and Toxicology, University of Regensburg, Regensburg, Germany

^2^ Institute of Physiology, University of Regensburg, Regensburg, Germany

^3^University of Virginia School of Medicine, Charlottesville, VA, USA

^4^ Institute of Pharmacology and Toxicology, Technical University of Munich, Munich, Germany

^5^ Bayer AG, Drug Discovery – Cardiology, Wuppertal, Germany

*** Correspondence:**Prof. Dr. Jens Schlossmann
[Jens.Schlossmann@ur.de](mailto:Jens.Schlossmann@ur.de)

# Supplementary Material

**Quantitative mRNA-analysis**

For mRNA-analysis, one kidney was excised and halved, stored overnight in a fridge in RNAlater (ThermoScientific, Braunschweig, Germany) for penetration into tissue and then transferred into -80°C until further processing. Total RNA was isolated from the kidney using the PeqGOLD TriFast^TM^ reagent (PeqLab, Erlangen, Germany) for phenol/chloroform extraction. After determination of concentration and quality control, 2 µg RNA was reverse transcribed (0.5 µg oligo-dT-Primer, 200 U M-MLV-RT, 20 U RNAsin) and 2 µl of cDNA were assayed for sGCβ1 (forward: 5’-TGCAAGCAAAGTCCTCAACCT-3’, reverse: 5’-ATCCCAGGACACGCAAGATG-3’) or PKGIα (forward: 5’-CGCCAGGCGTTCCGGAAGTT-3’, reverse: 5’-GTG CAG AGC TTC ACG CCT T-3’). GAPDH served as housekeeper (forward: 5’-CACCAGGGCTGCCATTTGCA-3’, reverse: 5’-GCTCCACCCTTCAAGTGG-3’) using the Roche LightCycler 480 system with SYBR green I (Roche, Mannheim, Germany) as fluorescent dye. For relative quantification the ∆∆Ct method was used; For absolute quantification, a standard curve of a dilution series of a known sample was recorded for each run and sGCβ1/GAPDH concentrations were hence calculated.

**Western Blot Analysis**

Proteins were extracted from whole kidneys in 2% lubrol (20 mM Tris, 140 mM NaCl, 2% nonaethylenglycolmonodecylether, 1 mM benzamidine, 0.5 µg/µl leupeptin, 0.3 mM PMSF, PhosStop, pH=8.0) using an ultraturrax and 50-70 µg of total protein was loaded on each lane. Following SDS-PAGE, proteins were blotted on a PVDF-membrane and incubated with soluble guanylate cyclase subunit β1 -antibody (ER-19; Sigma-Aldrich, Munich, Germany), PKGIα-antibody (Geiselhöringer et al., 2004) or PKGII-antibody (Schramm et al., 2014). Vinculin (R&D systems, Wiesbaden-Nordenstadt, Germany) or β-actin (abcam, Cambridge, UK) served as loading controls. Secondary antibodies were purchased from Dianova (Hamburg, Germany) and bands were detected with the ChemiDoc system (Bio-Rad, Munich, Germany). Quantification was performed using the ImageLab-software (Bio-Rad, Munich, Germany).

**Immunohistochemistry**

4 µm slices of perfusion-fixed (3% para-formaldehyd) paraffin embedded kidneys were stained for PKGI (Geiselhöringer et al., 2004), renin (either chicken anti-renin (Machura et al., 2009) or goat anti-renin (R&D systems, Wiesbaden-Nordenstadt, Germany), soluble guanylate cyclase subunit β1 (ER-19; Sigma-Aldrich, Munich, Germany) or α-smooth muscle actin (α-SMA; Beckman Coulter, Germany). Secondary fluorophore-coupled antibodies were obtained from Dianova, Hamburg, Germany. Fluorescence was detected using an Axiovert 200 microscope (objective: 20x; Zeiss, Jena, Germany) and analyzed using the axiovision software. Kidneys from at least 3 different animals were stained per treatment, whereof one representative staining is shown.

**Blood pressure measurement**

Systolic blood pressure of conscious mice was determined by tail cuff plethysmography (IITC life science, Woodland Hills, USA). Mice were trained for at least 3 consecutive days, after that, approx. 30 measurements were recorded and values were averaged over 5-10 representative readings.

# Supplementary Figures and Tables


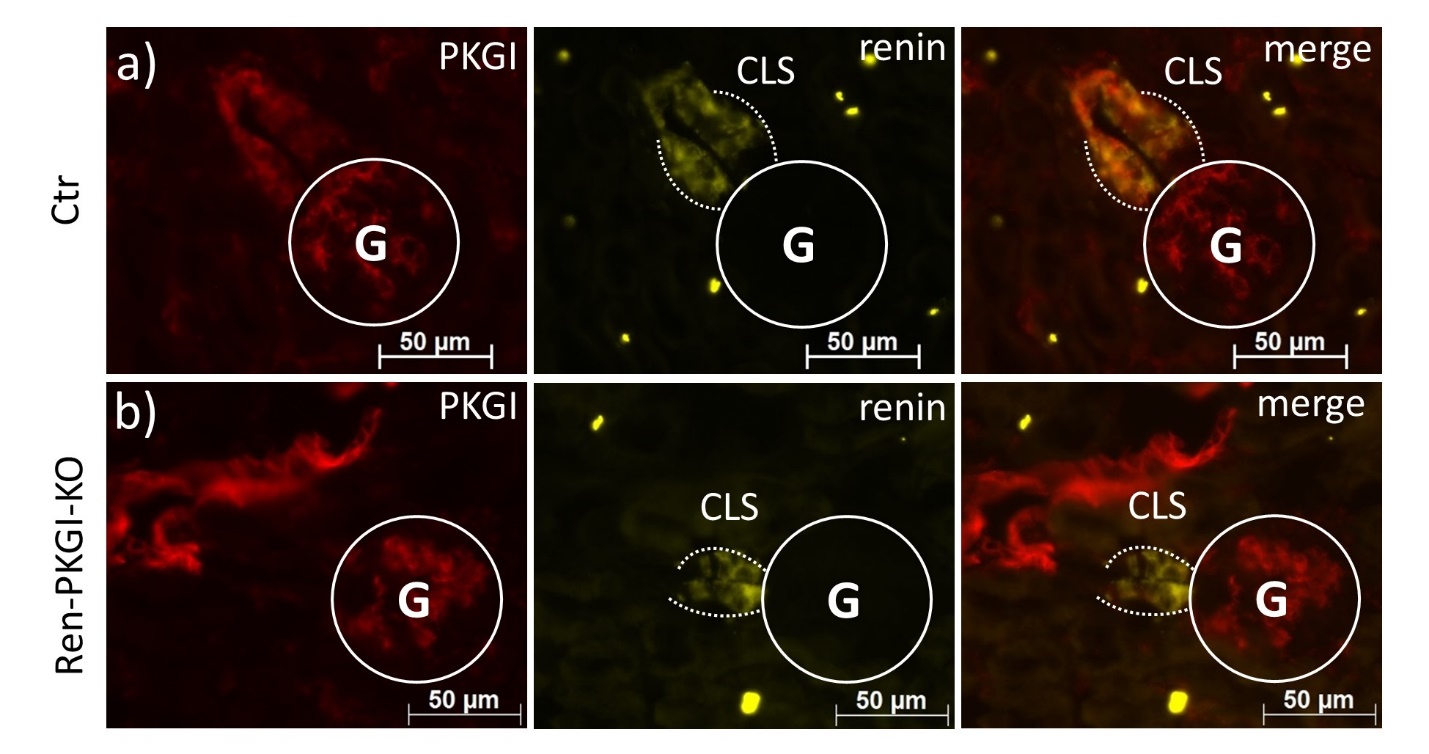


**Supplementary Figure 1: Demonstration of renin cells forming cuff-like structures following LS/Ena-treatment.**

Immunohistochemical Co-stainings from kidneys of (a) Ctr and (b) Ren-PKGI-KO animals after 3 weeks of LS/Ena treatment. Red: PKGI, yellow: renin. Dashed lines indicate hypertrophic areas (cuff like structures) formed after long-term stimulation of the RAAS. G = glomerulus, CLS = cuff-like structures.


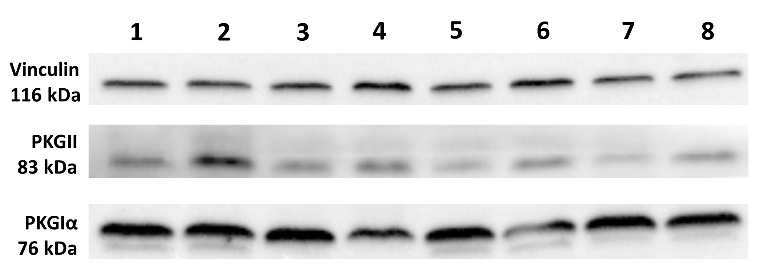


**Supplementary Figure 2: PKG abundance following different salt treatments:**

Renal PKGIα and PKGII protein expression of single conditional knockouts following different salt loads. Western Blot was loaded with 70 µg protein/lane and incubated with anti-vinculin (116 kDa) as loading control, anti-PKGII (83 kDa) and anti-PKGIα (76 kDa). Lane 1: Ctr, normal salt; lane 2: Ren-PKGI-KO, normal salt; lane 3: Ctr, low salt; lane 4: Ren-PKGI-KO, low salt; lane 5: Ctr, low salt+ enalapril; lane 6: Ren-PKGI-KO, low salt + enalapril; lane 7: Ctr, high salt; lane 8: Ren-PKGI-KO, high salt.


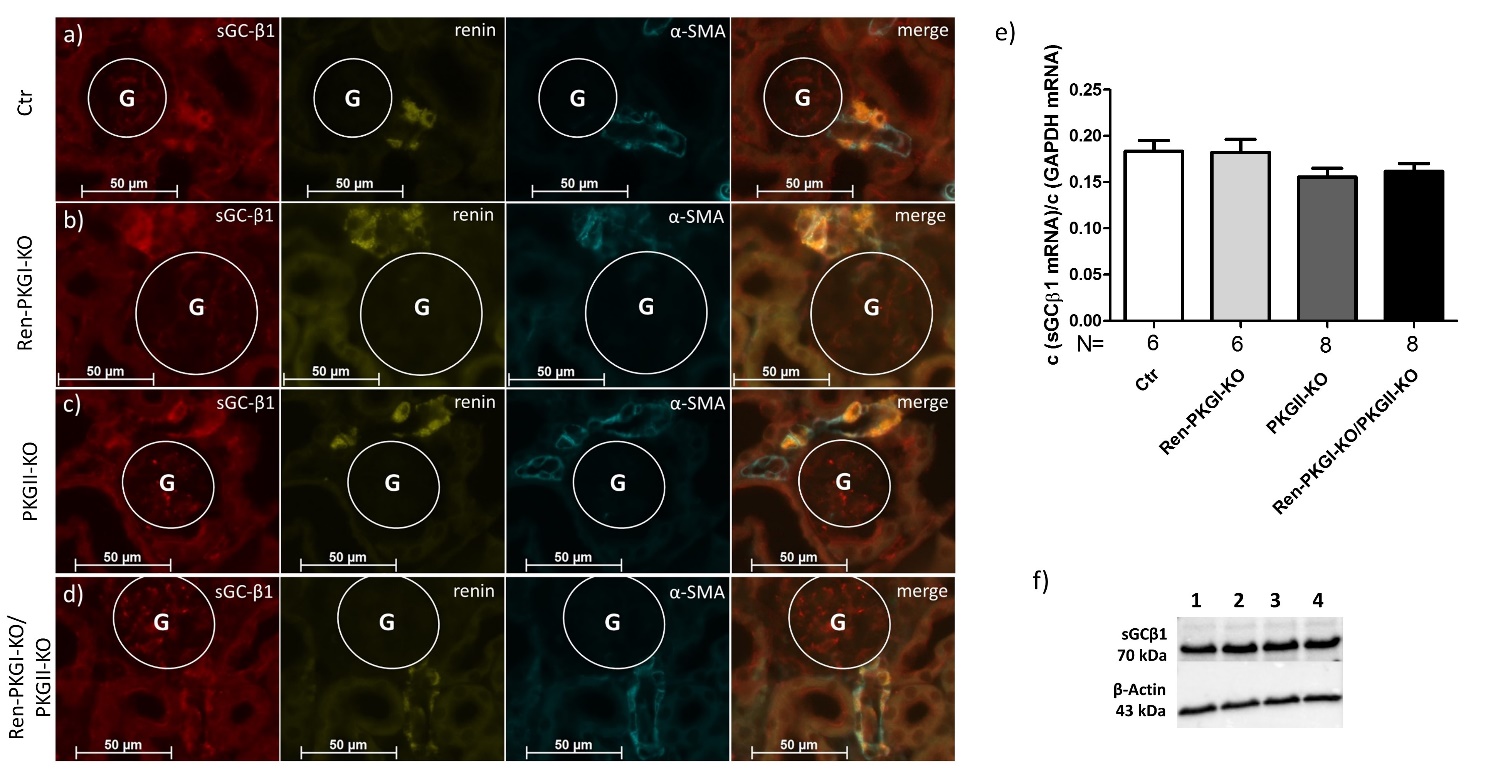


**Supplementary Figure 3: sGC analysis of kidneys of control -, single - and double knockout – animals receiving a normal salt diet:**

a-d) Representative co-stainings of soluble guanylate cyclase (sGC-β1, red) renin (yellow) and α-smooth muscle actin (α-SMA, turquoise). a) Ctr; b) Ren-PKGI-KO; c) PKGII-KO; d) Ren-PKGI-KO/PKGII-KO. Stainings were repeated at least 3 times from different animals. G = glomerulus.

e) sGC-mRNA, absolute quantification per standard curve, data is presented as mean ± SEM.

f) Representative Western Blot loaded with 70 µg protein/lane and incubated with anti sGC, subunit β1 (70 kDa) and anti-β-actin (43 kDa) as loading control. Lane 1: Ctrl; lane 2: Ren-PKGI-KO; lane 3: PKGII-KO; lane 4: Ren-PKGI-KO/PKGII-KO).

**
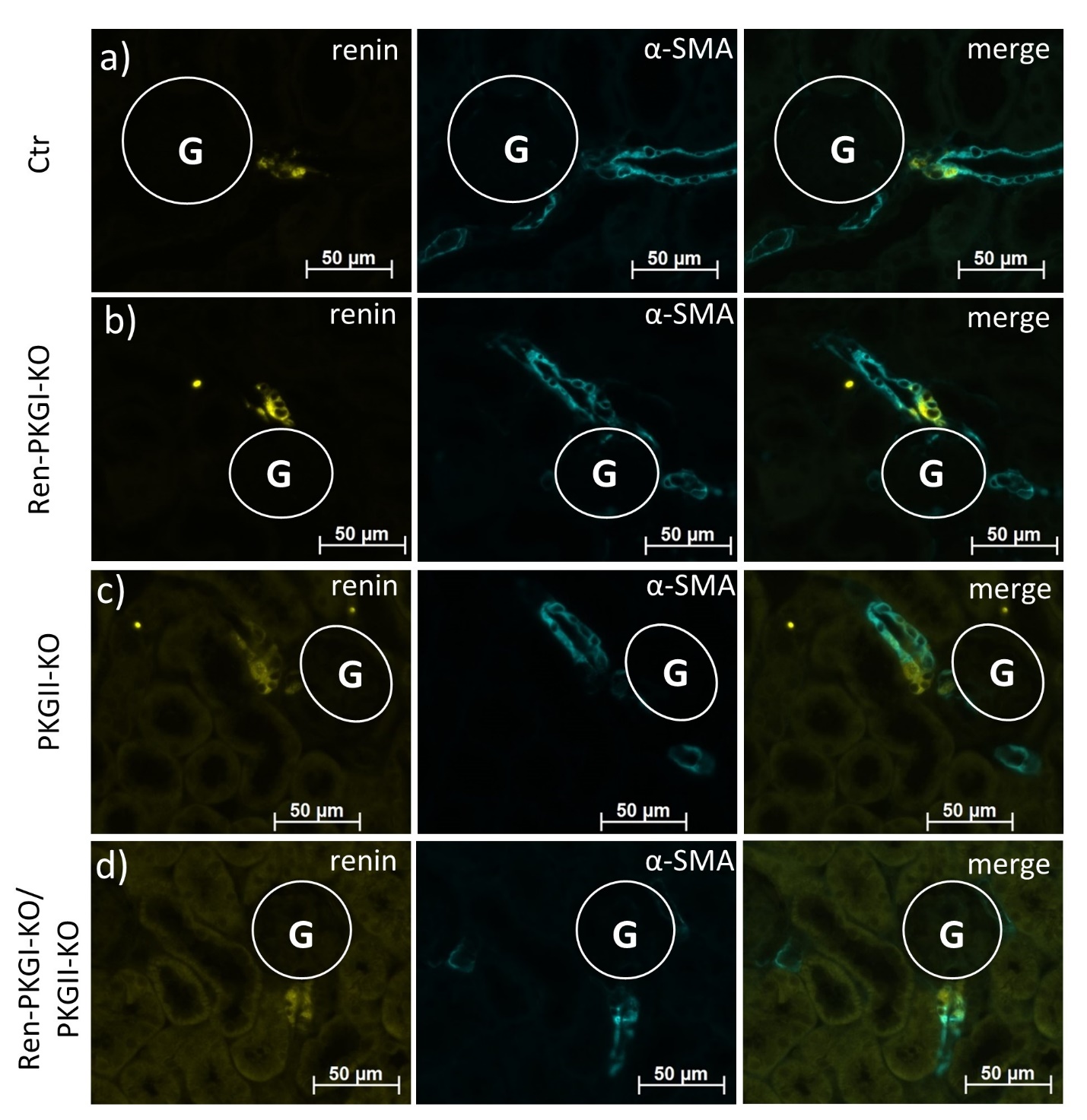
**

**Supplementary Figure 4: Absent recruitment following sGC stimulation:**

Immunohistochemical Co-stainings from kidneys of a) Ctr; b) Ren-PKGI-KO; c) PKGII-KO and d) Ren-PKGI-KO/PKGII-KO animals after 1 week of Bay 41-8543-treatment (1mg/kg, i.p.) with absent recruitment of transformed renin-producing cells distally from the glomerulus. Yellow: renin, turquoise: α-smooth muscle actin (α-SMA). G = glomerulus


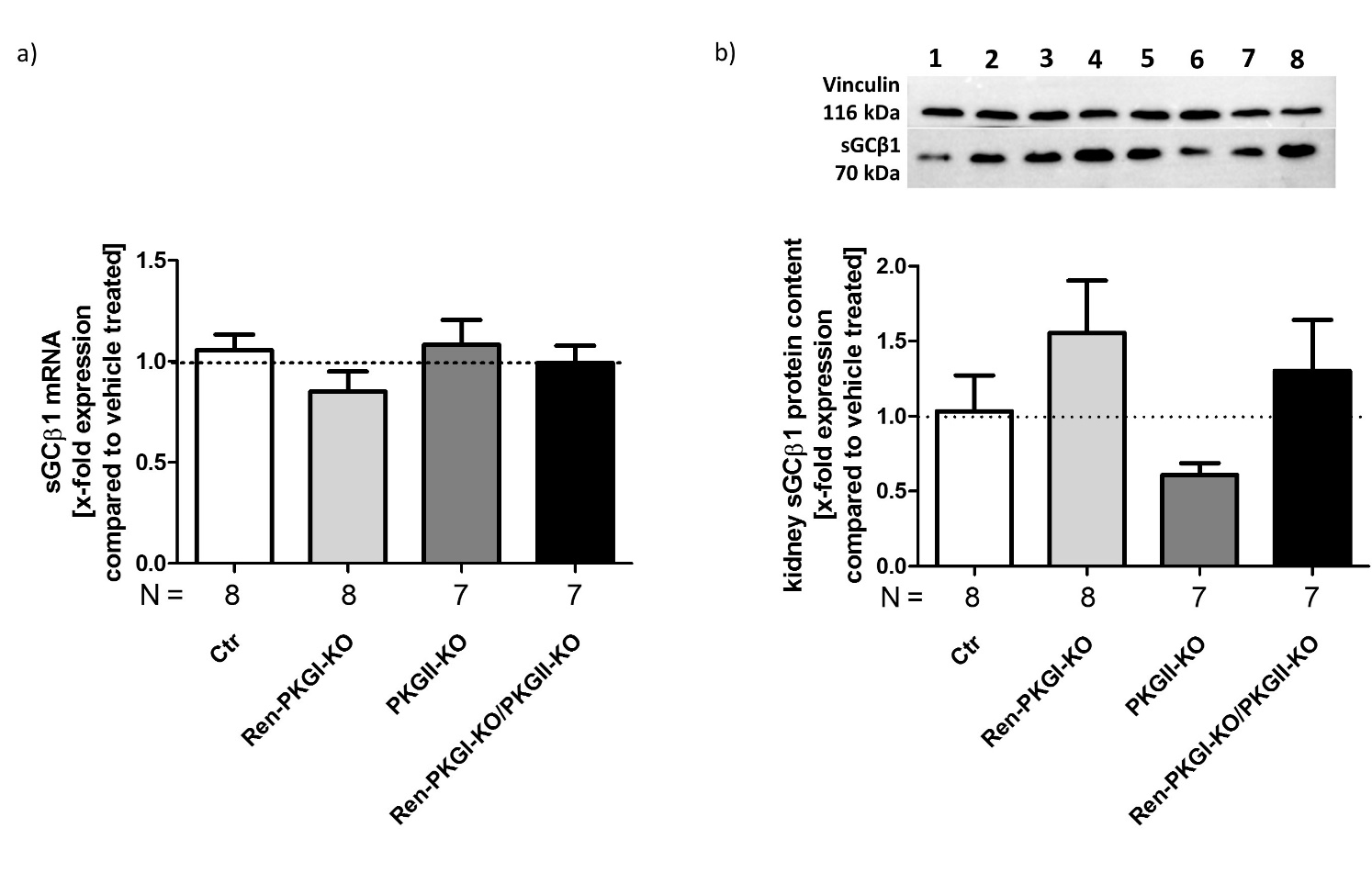


**Supplementary Figure 5: sGC abundance after chronic sGC stimulation:**

Mice were treated either with vehicle or with Bay 41-8543 (1 mg/kg BW) via intraperitoneal injection once a day for one week.

a: Renal sGC mRNA, relative quantification calculated per ΔΔCt compared to vehicle-treated animals of the same genotype.

b: Representative western blot and relative quantification of western blot data concerning renal sGC protein content. Western Blot was loaded with 70 µg protein/lane and incubated with anti-vinculin (116 kDa) as loading control and anti-sGC, subunit β1 (70 kDa). Lane 1: Ctr, vehicle-treated; lane 2: Ctr, Bay-treated; lane 3: Ren-PKGI-KO, vehicle-treated; lane 4: Ren-PKGI-KO, Bay-treated; lane 5: PKGII-KO, vehicle-treated; lane 6: PKGII-KO, Bay-treated; lane 7: Ren-PKGI-KO/PKGII-KO, vehicle-treated; lane 8: Ren-PKGI-KO/PKGII-KO, Bay-treated. Statistical analysis shows relative expression of sGC compared to vehicle-treated animals (Kruskal-Wallis-test).


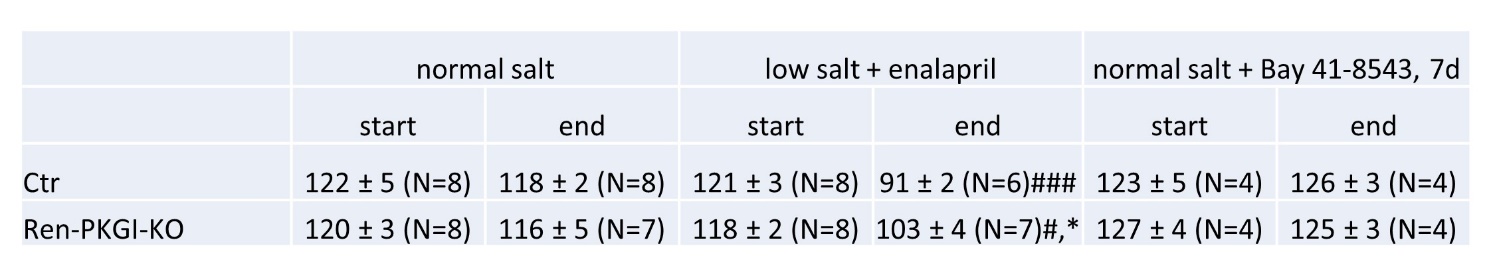


**Supplementary Table 1: Systolic blood pressure before and after different treatments:**

Systolic blood pressure was recorded before and after treatment with normal salt (NS) diet or low salt + enalapril (LS/Ena) for 3 weeks or before and after treatment with Bay 41-8543 (1 mg/kg BW) via intraperitoneal injection once a day for one week. Data is presented as mean ± SEM.

#,###: (highly) significant difference between LS/Ena treated mice vs. NS treated mice of the same genotype (ANOVA, p<0.05 for #; p<0.001 for ###).

* significant difference between LS/Ena treated Ren-PKGI-KO vs. Ctr animals (ANOVA, p<0.05).

Supplementary Literature

Geiselhöringer, A., Gaisa, M., Hofmann, F., and Schlossmann, J. (2004). Distribution of IRAG and cGKI-isoforms in murine tissues. *FEBS letters* 575, 19–22. doi: 10.1016/j.febslet.2004.08.030.

Machura, K., Steppan, D., Neubauer, B., Alenina, N., Coffman, T. M., Facemire, C. S. et al. (2009). Developmental renin expression in mice with a defective renin-angiotensin system. *American Journal of Physiology. Renal Physiology* 297, F1371‐1380. doi: 10.1152/ajprenal.00378.2009.

Schramm, A., Schinner, E., Huettner, J. P., Kees, F., Tauber, P., Hofmann, F. et al. (2014). Function of cGMP-dependent protein kinase II in volume load-induced diuresis. *Pflugers Archiv: European Journal of Physiology* 466, 2009–2018. doi: 10.1007/s00424-014-1445-y.
